# A T2* MRI prospective survey on heart iron in thalassemia major patients treated with deferasirox versus deferiprone and desferrioxamine in monotherapy

**DOI:** 10.1186/1532-429X-13-S1-O21

**Published:** 2011-02-02

**Authors:** Alessia Pepe, Antonella Meloni, Giuseppe Rossi, Maria Chiara Dell'Amico, Anna Spasiano, Marcello Capra, Paolo Cianciulli, Vincenzo Caruso, Brunella Favilli, Eliana Cracolici, Aurelio Maggio, Massimo Lombardi

**Affiliations:** 1 “G. Monasterio” Foundation and Institute of Clinical Physiology, CNR, Pisa, Italy; 2 A.O.R.N. Cardarelli, Napoli, Italy; 3 Ospedale "G. di Cristina", Palermo, Italy; 4 Ospedale "Sant'Eugenio Papa", Roma, Italy; 5 P.O. "S. Luigi-Currò", Catania, Italy; 6 Policlinico "Paolo Giaccone", Palermo, Italy; 7 Ospedale "V. Cervello", Palermo, Italy

## Background

No data are available about the efficacy of the new oral one daily chelator deferasirox on cardiac iron and function versus deferiprone and desferrioxamine. Magnetic Resonance (MR) is the unique non invasive suitable technique to quantitatively evaluate this issue.

## Aims

Our aim was to prospectively assess the efficacy of the deferasirox versus deferiprone and desferrioxamine in monotherapy in a large cohort of thlassemia major (TM) patients by quantitative MR.

## Methods

Among the first 1135 TM patients enrolled in the MIOT (Myocardial Iron Overload in Thalassemia) network, 392 patients performed a MR follow up study at 18 ± 3 months according to the protocol. We evaluated prospectively the 193 TM patients who had been received one chelator alone between the 2 MR scans. We identified 3 groups of patients: 80 treated with DFX, 39 treated with DFP and 74 treated with DFO. Myocardial iron concentrations were measured by T2* multislice multiecho technique. Biventricular function parameters were quantitatively evaluated by cine images.

## Results

Excellent/good levels of compliance were similar in the 3 groups (DFX 99%, DFP 95%; DFO 96%, *P* = 0.6). There were no significant differences in all 3 groups to maintain the patients without significant myocardial iron overload (global heart T2*≥20 ms) (DFX 98%; DFP 100%; DFO 98%; P=1.0). The percentage of patients that maintained a normal LVEF (>57%) was significantly lower in DFX (77%) versus DFP (100%) (P=0.018), it was no significantly different in DFX and DFO group (82%) (P=0.59).

**Figure 1:**
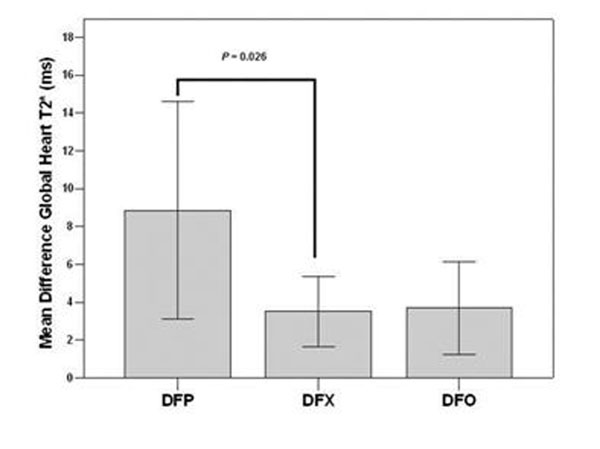


Among the patients with myocardial iron overload at baseline in all 3 groups, there was a significant improvement in the global heart T2* value and in the number of segment with a normal T2* value; only in the DFP group there was a significant improvement in the right global systolic function (+ 6.8% *P* =0.016). The improvement in the global heart T2* was significantly lower in the DFX versus the DFP group (*P*=0.0026), but it was not significantly different in the DFX versus the DFO group (mean difference global heart T2* 3.5±4.7 ms versus 8.8±8.6 ms and versus 3.7±5.5 ms, respectively; P=0.90) (Figure 1). The changes in the global systolic bi-ventricular function were not significantly different among groups.

## Conclusions

Prospectively over 15 months in a large clinical setting of TM patients DFP monotherapy was significantly more effective than DFX in improving myocardial siderosis and in maintaining a normal LVEF, no significant differences were found between DFX and DFO monotherapy.

